# Healthcare professionals’ views of the enhanced recovery after surgery programme: a qualitative investigation

**DOI:** 10.1186/s12913-017-2547-y

**Published:** 2017-08-31

**Authors:** Georgia Herbert, Eileen Sutton, Sorrel Burden, Stephen Lewis, Steve Thomas, Andy Ness, Charlotte Atkinson

**Affiliations:** 1 The NIHR Biomedical Research Unit at the University Hospitals Bristol NHS Foundation Trust and the University of Bristol in Nutrition, Diet and Lifestyle, Bristol, UK; 2 School of Nursing, Midwifery and Social Work, University of Manchester, Manchester, UK; 3 Derriford Hospital, Plymouth, UK; 4 Oral and Maxillofacial Surgery, University of Bristol, Bristol, UK

**Keywords:** Enhanced recovery after surgery Programme, Qualitative, Normalisation process theory, Healthcare professionals

## Abstract

**Background:**

The Enhanced Recovery After Surgery (ERAS) programme is an approach to the perioperative care of patients which aims to improve outcomes and speed up recovery after surgery. Although the evidence base appears strong for this programme, the implementation of ERAS has been slow. This study aimed to gain an understanding of the facilitating factors and challenges of implementing the programme with a view to providing additional contextual information to aid implementation. The study had a particular focus on the nutritional elements as these have been highlighted as important.

**Methods:**

The study employed qualitative research methods, guided by the Normalisation Process Theory (NPT) to explore the experiences and opinions of 26 healthcare professionals from a range of disciplines implementing the programme.

**Results:**

This study identified facilitating factors to the implementation of ERAS: alignment with evidence based practice, standardising practice, drawing on the evidence base of other specialties, leadership, teamwork, ERAS meetings, patient involvement and education, a pre-operative assessment unit, staff education, resources attached to obtaining The Commissioning for Quality and Innovation (CQUIN) money, the ward layout, data collection and feedback, and adapting the care pathway.

A number of implementation challenges were also identified: resistance to change, standardisation affecting personalised patient care, the buy-in of relevant stakeholders, keeping ERAS visible, information provision to patients, resources, palatability of nutritional drinks, aligning different ward cultures, patients going to non-ERAS departments, spreading the programme within the hospital, differences in health issue, and utilising a segmental approach.

**Conclusions:**

The findings presented here provide useful contextual information from diverse surgical specialties to inform healthcare providers when implementing ERAS in practice. Addressing the challenges and utilising the facilitating factors identified in this study, could speed up the rate at which ERAS is adopted, implemented and embedded.

## Background

In 2009, the Department of Health in England set up the Enhanced Recovery After Surgery (ERAS) programme and its use has been encouraged in NHS hospitals across the United Kingdom [1]. ERAS is an approach to the perioperative care of patients which encompasses multiple evidence-based interventions on the basis that these will improve patient outcomes and speed up their recovery after surgery [2]. The programme includes the whole patient journey from referral to post-surgical follow-up, incorporating around 20 components.

Studies have consistently shown that implementation of ERAS has positive outcomes in terms of both hospital length of stay and complications [3]. Although the evidence base appears strong for this programme, the implementation of ERAS has been slow [2]. To use the ‘efficacy knowledge’ [4] that surrounds ERAS, and translate evidence from trials into routine clinical practice, decision makers require contextual information on effectiveness in different settings, and guidance on implementation [5, 6]. In order to facilitate this, qualitative work is key.

The lack of qualitative information to guide the most effective implementation could provide some rationale for the lack of engagement with this programme [2, 7]. Furthermore, the existing qualitative research base is limited to colorectal surgery outside of the UK [8–12], and although some principles may be considered generalizable and translate to the UK and other surgical specialties, difficulties can occur when guidelines are transferred from one context to another [13]. A recent synthesis suggests that further information is required on how ERAS is implemented and experienced in NHS settings [14].

The nutritional elements within the programme (avoidance of long periods of preoperative fasting, the use of preoperative carbohydrate loading and re-establishment of oral feeding as soon as possible after surgery) are of particular interest because patients have highlighted food as the most important area for service improvement [15]. Furthermore as there has been a radical change in practice implementation in this area, the dietary elements within the programme could be deemed as more challenging [13, 16, 17].

The aim of this study was to gain an understanding of the facilitating factors and challenges of implementing an ERAS programme within a UK context in three different specialities: colorectal, head and neck and thoracic with a focus on the nutritional elements.

## Methods

### Study design and recruitment

The study employed qualitative research methods, guided by the Normalisation Process Theory (NPT) to explore the experiences and opinions of health care professionals implementing an ERAS programme. Twenty-six semi-structured interviews were conducted with health care professionals in a regional teaching hospital; twenty-one interviews were conducted with those working across three wards (colorectal, head and neck and thoracic), and five with those working in roles that cut across these specialities (Table 1).

**Table 1:** Participant characteristics

Speciality	SUR/ANS	NUR/AHP	CLINMAN/MAN	Total
Cross-cutting	0	3	2	5
Thoracic	4	2	1	7
Colorectal	2	4	1	7
Head & Neck	3	3	1	7
Total	9	12	5	26

*SUR/ANS* Surgeons and Anaesthetists, *NUR/AHP* Nurses and Allied Health Professionals (Dietitian, Physiotherapist, Speech & Language Therapist) and Housekeeper, *CLINMAN/MAN* Clinical Managers (including Ward Managers and Ward Sisters) and Trust management, *Cross-cutting* Roles that cut across specialities

Selection of the three surgical areas was guided by the desire to qualitatively explore experiences of ERAS implementation from other specialities alongside those of colorectal. Interviews were conducted by GH and ES, audio-recorded with participants’ written consent and transcribed verbatim by an approved transcription service. Interviews lasted between 23 and 78 min (mean 41) and were guided by an interview guide developed from a review of relevant literature with topics for discussion structured around the four main constructs of Normalisation Process Theory [18].

Participants were recruited by means of an initial invitation email sent to surgeons already known to the research team. Other healthcare professionals were then recruited by snowball-sampling as each participant was asked to recommend another member of staff who they felt played a pertinent role in the ERAS programme. Interviews continued until a range of professionals had been consulted and data saturation had been reached when no new themes were emerging during the interviews.

### Data analysis

Analysis was carried out with the aid of the NVivo (Version 10) software package using an adapted Framework Approach [19]. An initial coding framework was developed structured around the four main constructs of NPT [18]. Transcripts were independently coded by both GH and ES to ensure robustness of the data coding process as they conferred regularly to revise the coding framework. Descriptive accounts of the main constructs and sub-constructs within these were then developed. The analysis process compared and contrasted data both across and within specialities and disciplines in order to better understand both facilitating factors and challenges to implementation.

### Normalisation process theory

The NPT is a theoretical model that provides an explanatory framework to help understand how practices are implemented, embedded, and integrated into their social contexts [20]. NPT has been used across a range of healthcare contexts [21–24] and has been successfully employed in the analysis of the implementation of complex interventions [25]. It provides a framework for investigating the collective work that may or may not lead to a programme being incorporated into everyday work. NPT suggests implementation of an intervention is operationalised through four main constructs: the theory helps us to understand the way people make sense of the work of implementing and integrating an intervention individually and as a team (construct 1: coherence); how they engage with that intervention (construct 2: cognitive participation); how they enact it (construct 3: collective action); and appraise its effects (construct 4: reflective monitoring) [18]. NPT was used in the development of the interview topic guide and to aid analysis of interview data.

## Results

The findings are presented within the four main constructs of the NPT framework, together with illustrative quotations, a summary of which can be found in Table 2.

**Table 2:** Facilitating factors and challenges of implementing an ERAS programme by NPT construct

NPT construct	Facilitating factors	Challenges
Coherence	- Alignment with evidence based practice - Standardising practice – incorporation into routine activity - Drawing on evidence base in other specialties^a^	- Resistance: Breaking down entrenched surgical dogmas^a^ - Standardisation affecting personalised patient care
Cognitive Participation	- Cohesive, visible leadership amongst surgeons and nurses - Teamwork – engagement of all relevant stakeholders - ERAS meetings	- Buy-in of relevant stakeholders - Keeping ERAS visible
Collective Action	- Patient involvement and education – ERAS diaries^a^ - Pre-operative assessment unit - Staff education^a^ - Resources attached to obtaining CQUIN money (e.g. data collectors, ring fence nursing time) - Ward layout – protected beds	- Information provision to patients – volume affecting retention - Resources - Staff (being short staffed, high turnover, lack of weekend workers)^a^ - Lack of time (to attend meetings, educate staff and patients) - Lack of management support - Ending (e.g. money attached to CQUINs) - Nutritional drinks – palatability^a^ - Merger – aligning different ward cultures - Patients going to non-ERAS departments – ICU^a^ - Spread within the hospital - Health issue^a^ - Segmental approach
Reflective Monitoring	- Data collection and feedback - Adapting the care pathway	

^a^Nutritionally related

### Coherence – Making sense of the ERAS programme

#### Evidence and standardization

The existing evidence base for the programme was generally seen as legitimate and all participants understood ERAS to have benefits, for example, in reducing length of stay, complications, and enhancing patient experience. Participants, especially surgeons and anaesthetists, were keen to emphasise the importance of alignment with evidence-based practice when implementing the programme:

*…so there is a decision making that happens as evidence-based as possible. (SUR/ANS-TH-14)*

*…it’s starting off by taking an evidence based approach to what we do, so protocolising with as much care as we can (SUR/ANS-TH-13)*

The nutritional aspects of ERAS across the specialties (no prolonged fasting, pre-operative carbohydrate loading and post-operative early feeding) were reported to be important and “make sense” (SUR/ANS-TH-16). Some interviewees within the thoracics specialty mentioned a lack of evidence with regard to the ERAS elements relating to nutrition. However, this didn’t deter the implementation of these elements, and the nutritional status of the patient was considered important. In the absence of evidence, they looked to the evidence base surrounding other specialties.

*So it kind of makes intuitive sense that we should feed these patients. Erm, but we don’t really have an evidence base for that, but because the pathways looked appropriate in colorectal, we just essentially copied and pasted, and the same with pre-op carbohydrate loading. Well, it makes sense not to have your patients dehydrated. (SUR/ANS-TH-16)*

However, setting up and enacting ERAS practices were not always smooth processes. Some participants reported encountering resistance from colleagues, and in a few cases, especially within the colorectal speciality, described the need to break down entrenched surgical dogmas with regard to feeding practices:

*…we’ve had a lot of resistance. Clinical colleagues, consultant colleagues - a few yes and a few no – “I've done it for 20 years, why should I do anything different?”(*SUR/ANS-CO-20*)*

ERAS was seen as a trigger to the standardisation of care along evidence-based lines. It enabled the standardisation of certain aspects of work, for example, in the case of nutritional screening, which was not previously organised, ERAS was thus described as “*a vessel for change*” (NUR/AHP-CC-4), improving patient care. Standardising practice was considered important to help overcome inconsistencies in patient care:

*So I think protocols are the way forward to do these things, because … a patient comes in on a Monday, Tuesday and Wednesday for the same operation, they should get the same service irrespective of whether I am there or not, because we should all be doing the same thing. (SUR/ANS-TH-16)*

Those within the thoracics speciality, reported that the distribution of nutritional drinks (e.g. Fortisips) three times a day had been logistically challenging as other aspects of patient care could take priority. The team protocolized this element of ERAS and incorporated it into a routine activity, which facilitated its implementation.

*But what we do now, it’s just built into our normal daily routine is that we do the Fortisip drink rounds. So someone will put all the drinks on a trolley three times a day and walk around the ward, and offer [them to] patients. (NUR/AHP-TH-17)*

For some, protocols were considered to be a tool to “nudge” (SUR/ANS-TH-16) them to carry out particular components of ERAS. One individual spoke about the protocol as providing the means to challenge practice that deviated from agreed actions:

*We had a patient where, erm, the tracheostomy protocol wasn’t followed … it was quite useful to actually be able to, erm, say, you know, to the consultant who was involved in that, “Look. This actually is something that we agreed and this wasn’t followed” … we felt like we had some evidence, as a consensus view that, you know, to, to challenge, erm, practice that wasn’t following standard practice. (NUR/AHP-HN-9)*

Although standardisation was generally viewed positively by participants, a few voiced concerns that ERAS had turned into a ‘tick-box’ exercise and that sticking to protocol monitoring too rigidly absorbed time better given to personalised patient care:

*I think the barrier there is just too much documentation and not enough onus on physically giving the care to the patient. You can spend an hour just filling in a care plan, where that hour could be even just talking to a patient, “How are you feeling”. (NUR/AHP-HN-12)*

Some participants stressed the need for flexibility in implementing protocols in practice to ensure a desired level of clinical autonomy so individual patient needs were met:

*It’s very much based on the patients, and I think we, as much as the patients have their goals from a mobility point of view it’s, “You will sit out for two hours four times a day, and you’ll walk 60 meters once today, and tomorrow you’ll do it twice.” We ignore that completely and basically go on each patient and their functional capabilities. (NUR/AHP-CC-3)*

### Cognitive participation – Investing in the ERAS programme

#### Buy-in and maintenance

Engagement with the ERAS programme was attained through both bottom-up influences of enthusiastic clinicians who evolved into ERAS leads or ‘champions’ for the programme, and top-down pressure from the trust. However, securing buy-in to the programme was a commonly reported challenge:

*I think it can be quite a thankless task at times doing this sort of work … you can take a horse to water, but you can't make it drink. And that's what it feels like sometimes. (CLINMAN/MAN-CC-1)*

*One of the problems, um, we’ve had is engagement of the nursing group as a whole. (SUR/ANS-HN-7)*

Once commitment had been established, consolidating enthusiasm for the programme and keeping ERAS visible was another of the main challenges:

*There’s only so much nagging of a team that you can do … People can’t absorb information constantly … And put it all into practice. It’s difficult. (NUR/AHP -TH-19)*

*This job has made me realise that - my own ward I used to manage how if you don’t enforce it they will forget. (NUR/AHP-HN-12)*

### Leadership and teamwork

Cohesive, visible leadership of the programme amongst the consultant medical staff was considered to be a key facilitating factor for successful implementation:

*I think the most important person to have really signed up and really driving it forward is a consultant surgeon who’s taking the lead for a particular area. (NUR/AHP-CC-4)*

Having leadership at the nursing level was reported as being equally important to be able to drive the programme forward on the ward. The vision for many was for ERAS to be nurse-led:

*Arguably, just as important, from the nursing perspective, is making sure that you’ve got senior members of the nursing team that are able to sort of push it forward, as well. Because … the day-to-day running of ERAS is very much down to the nursing staff on the ward. (NUR/AHP-HN-10)*

*.. it would be nice if overall nurses would realise that this is something that they deliver and it’s extremely important for the patients’ recoveries. It’s probably more important than the surgery itself. Um, and they should take it as an ownership of it. (SUR/ANS-TH-14)*

One individual described how they felt implementation in their speciality was restricted because the programme hadn’t been surgeon or nurse-led:

*… but I think it is an issue having two anesthetists running it because actually the vast majority of what’s required is actually the ward stuff, and it’s hard for us to take leadership of the ward stuff. It really needs to come either from the nurses or from the surgeons, erm, so, I think that’s partly why I feel we’ve stalled at the moment because …the bit that we’re much more involved in which is the in-theatre bit and the pre-op assessment bit, well, that was, kind of, already in place anyway. (SUR/ANS HN-8)*

Whilst a few suggested that it was key to have one person to focus enthusiasm and push implementation forward, many reported that implementation had been stymied when such an individual had left and the skills and support they offered had not been replaced. A “centre pin” (SUR/ANS-TH-16) approach was not considered conducive to sustaining implementation efforts:

*I think there’s certain key things that need to be addressed … key boxes that need to be ticked by an enhanced recovery programme to make sure it is sustainable … so it can’t be reliant on one individual or one role, because – well, for obvious reasons. If you take that person out of the equation then the whole thing will come crumbling down. (NUR/AHP-CC-4)*

Instead, participants described the engagement of all relevant stakeholders, teamwork and collaboration as critical facilitators for successful programme implementation:

*I think it should be led by the team really. I think it’s one of those things that someone can initiate it and someone can start to lead, but if the team doesn’t take over then it’s probably doomed. (NUR/AHP-TH-19)*

#### ERAS meetings

Meetings that were focused on ERAS implementation within each of the specialities were considered an important part of the process of developing an ERAS programme as they brought together the multidisciplinary team at the programme set-up stage. It meant that all stakeholders had a chance to shape the planned work, as one surgeon stated “they were there to invent it” (7, HN). Participants described the meetings as offering a rare and much appreciated opportunity to spend time with colleagues to reflect on practices, address differences, standardise and clarify processes, and to discuss ways to go forward with the programme as a team:

*I think the ward staff and the, allied health professionals and the surgeons having a chance to sit down and talk through what they each thought was going on, which was not always the same (Laughter)…was quite a useful process in itself. So it was really about you know, streamlining and, clarifying what was going on. (SUR/ANS-HN-8)*

### Collective action – Implementing the ERAS programme

#### Available resources

Healthcare professionals across the specialities and disciplines considered staff education to be an essential but challenging element of ERAS implementation.

*…you’re trying to educate a very busy group of people, and that can be difficult. They’re not the type of people you can say, “Right, everyone that works here, drop off your Wednesday afternoon, and I’ll come and teach you.” Because it just doesn’t work like that. Erm, everyone’s got patients to look after, and you know, the fundamental problem with educating a group of people that are here 365 days a year, 24 hours a day, like nurses, is that you can never get them all in one place. (NUR/AHP-CC-4)*

Education of staff was necessary to encourage the early feeding element of ERAS:

*…and to educate the nursing staff on the ward, erm, and generate the consensus among my colleagues that, erm, we can look after the patients according to the ERAS protocol and not just starve them for three days. (SUR/ANS-CO-21)*

Resource limitations in terms of staffing issues (being short staffed, high staff turnover, lack of weekend workers including dietitians) and time constraints (to attend meetings, educate staff and patients) were also reported as significant challenges to implementing the ERAS programme by many of the participants across roles:

*That's one area which I think we can work better because some patients get stomas and I think that delays their ERAS a little bit because we don't have enough staff or manpower to educate them about stomas pre-operatively. (SUR/ANS-CO-21)*

*It was, very much from my perspective … “we’ve implemented ERAS, these meetings have gone on, we decided that’s what we’re going to do, we’ve got the booklets and then let’s roll with it.” “So tell as many nurses as possible,” but we didn’t have the time to actually tell them in-depth what it means. (NUR/AHP-HN-12)*

The role of money attached to CQUIN scheme was recognized by many staff as key to facilitating implementation, as successfully reaching ERAS-related targets provided the funds for additional resources. For example, project nurses time was back filled, extra equipment and data collectors were available. Staff in the thoracics speciality described having used CQUIN money to ring-fence nursing time dedicated to the implementation of the ERAS programme. Many considered this to have enabled ERAS implementation as the allocated time had maintained ERAS focus and enthusiasm:

*…certainly early on there was a drift in – it was introduced and everyone was signed up to it and then it drifted back a bit … And so my feeling was when the two ERP nurses came, they were already on the ward, but were appointed into that role, I think they were very good at keeping it ticking over with the nurses … (SUR/ANS-TH-13)*

*…there was some bit of protected time given to some of the thoracic staff to take some time out and that’s how it got so micro-managed and how it got so embedded in thoracics. (NUR/AHP-CO-24)*

However, a few participants reported that it was challenging when project-associated resources ended:

*…it’s more difficult now because we don’t get allocated time. So everything’s done on the run, whereas when we were doing the project nurse we were given specific hours. (NUR/AHP-TH-18)*

Despite senior management asserting support for the programme e.g. through a transformation programme, this support was not always experienced by clinicians:

*I think the Trust implementation, they think it’s a great idea … implementation is one thing, follow up is another and, actually, this sort of work isn’t being done by the Trust … and so, it’s sort of setting off on, um, potentially a tick box exercise if there is nobody actually following it up and, you know, removing the barriers. So I think that is a barrier, is the Trust’s real involvement. (SUR/ANS-HN-6)*

Participants felt a lack of support was demonstrated by the failure to replace key staff or provide staff such as data collectors, which challenged implementation as they could not receive feedback on the success of their efforts:

*I'm a bit frustrated at the moment because I feel a bit let down by the Trust, in that, you know, everybody’s worked very hard to get this up and running and about the only thing the Trust themselves needed to do on the management side was, you know, provide the data collector and they’ve failed (Laughter) … and then everybody just gets a bit, sort of, er, “That was all a bit of a waste of time,” and then it’s - that’s really difficult to try and keep the momentum. (SUR/ANS-HN-8)*

One individual in a management position was aware that their support may not have been recognised:

*I'm not sure the teams on the ground would feel that it's absolutely something that's being really supported. And just as I'm sitting here … I can't tell you exactly how much we're doing and that frustrates me … if the clinical teams were able to find a really clear way of doing it, and the, er, senior manager find a really clear way of, erm, expressing their support for it we may be able to move forward faster and better, you know, and some of that translating, then, into reality on the ground as well, and it's not, it's not left - just our fine words. What does that mean in terms of the teams getting what they need, and I guess, you know, senior managers getting what they need? (CLINMAN/MAN-CC-2)*

#### Patient involvement and education

For the successful implementation of ERAS, staff reported the need for patients to “buy-in to” (NUR/AHP-CO-24) the programme, which required them to take an active role in their own recovery. This was described as patient empowerment, or as them signing up to a “contract of care” (NUR/AHP-CC-3). The shift from ‘sick patient’ to ‘empowered patient’ was considered as an important culture change, and was viewed by some as a significant driver to ERAS success:

*I think it's just a culture change … and empowering our patients much more. That, actually, it's okay to go home after four or five days. You know, it's not because we don't care, and it's not because we're a bad organisation or [provide] shoddy care… Actually, it's the right thing for you to be recovering in your own home (CLINMAN/MAN-CC-1)*

Education of the patient, through face-to-face clinical contact and the provision of good quality information, was viewed as a key facilitator to this change by most of the participants interviewed:

*... patient expectations of coming into hospital and being poorly aren’t what we want them to have. We want to re-educate their expectations … (NUR/AHP-CO-24)*

*So, it’s actually listening to patients and, um, changing their expectations and not institutionalising people. (SUR/ANS-HN-6)*

For example, the palatability of the nutritional drinks that patients were required to drink before and after their operation was reported as a challenge. Healthcare professionals across the specialties and disciplines described patients finding the nutritional drinks difficult to “tolerate” (NUR/AHP-CO-24) especially in the post-operative phase. Encouragement and education from ward staff was considered important for patients to consume the drinks:

*If they are day two or day three post-op and you do a drug round and you say, “You are due for your nutritional drink.” They will say, “Actually I don’t want it.” They’ve had enough of it by then … And I think they are quite difficult to tolerate if you’re not feeling brilliant. (NUR/AHP-CO-24)*

*I think initially, people were wondering why they had to have … the Fortisip drinks three times a day, especially when they didn’t like them. Um, so it’s just educating people that, you know, although they’re eating well, there’s just a little bit of a nutritional boost because post-surgery they need a little bit of extra. So once they knew that it wasn’t just something we wanted to give them like a gimmick, that it was actually- they were a lot happier to take it on board. (NUR/AHP-TH-18)*

The pre-operative assessment unit was reported to be integral to educate the patient. Providing patients with information about carbohydrate loading, nutritional supplementation, nutrition post-surgery and early mobilisation was part of a process of ‘patient optimization’ so that they would be in the best possible condition on the day of surgery and also aid their recovery post-surgery. However participants were concerned about the volume of information provided to patients prior to surgery, the problems of information retention and ERAS-specific information getting lost:

*There’s significant variation with individuals as to how well they’re able to retain that information, as well. You know, they’ll be given such an enormous amount of information prior to coming in … that the enhanced recovery side of things is something that can sometimes get a little bit overlooked. (NUR/AHP-CC-4)*

ERAS diaries were given to patients which set out the steps they should aim to achieve as preparation for surgery and throughout their recovery. Many providers thought they facilitated implementation as they were a tool to educate, empower and motivate the patient to take an active role in their care, including their nutritional intake:

*My view is the patient diaries are a way to motivate the patients to keep on track and to remind everybody else like the nursing staff and the doctors “Well, shouldn't my catheter be coming out today?” “Shouldn't my drain, I've got a drain, why have I got a drain?” “Shouldn't my epidural be coming down?” “Why aren't I allowed to eat, why aren’t I eating, where's my nutritional drinks?” “I need to do my 50 yard walks”…It's them to motivate the patients and them to question if it's not happening which I think is the best way, it's empowering and motivating a patient. (SUR/ANS-CO-20)*

Some reported the problem of patients failing to bring their ERAS diaries on admission to the ward, and a few had *“mixed feelings”* (NUR/AHP-HN-9) towards them because some patients might be discouraged if they were not making good progress along the pathway.

#### Environment/organisational structure

Another factor that was reported as important to consider when implementing ERAS was the physical geography of the wards. Communal areas such as separate dining rooms and nutritional drinks fridges where individuals could help themselves to their post-operative nutritional drinks was thought to empower the patient:

*We’ve concentrated patient care to their bedside and so there needs to be an encouragement to move away from that. So you need communal areas, you need areas where the patients can meet in the corridor and they can chat in the corridor, you need access to extra nutrition so for example when we went to [name of city] …, the wards had occasional tables down the ward with two chairs at each table and a bowl of fresh fruit, there was a buffet area so if the patients could get up, then they got up and ate in the buffet area and relatives were allowed on the ward to eat with them …everything was aimed at trying to create an environment which wasn’t like being on a long-haul flight, it was more like being at home … that’s what you need …to get you better quicker. (SUR/ANS-TH-15)*

Space was important; having bathrooms that were large enough to turn around with a drip encouraged early mobilisation. The positioning of fridges stocked with nutritional drinks was an issue for some:

*Twice a day, up you get, you go walking … with your nurse and you go and help yourself to your drinks. But you see on here we got a fridge here, I’ve got a fridge in one of our store rooms. There’s no space, the corridors are meant to be clear, where do you put the fridges? (CLINMAN/MAN-CO-22)*

Participants reported that homogeneity of patients on a ward, and having protected beds for non-emergency ERAS patients facilitated implementation.

*…the sort of golden standard would be to have ring fence protected beds. And like I said, it worked for a couple of months I think, and then just with the pressures of beds in general and the capacity it went by the wayside. So a decision was made to change. It was made at senior management level. It was nothing to do with us, um, at ward level, but it was just that the hospital had to look at the bigger picture rather than just the enhanced recovery picture, which is a shame. (NUR/AHP-TH-17)*

During the fieldwork period for the colorectal ward, environmental changes were taking place in the Trust with the merging of wards across the specialisms. This was considered to have tested the implementation and embedding of ERAS. One of the biggest challenges for staff was aligning different ward cultures as wards merged. Nurses and ward managers reported the merger had created greater diversity on the wards for them to navigate in terms of the conditions and ERAS-status of the patient (whether the patient was on ERAS), and the paperwork and systems in use:

*It has been a big challenge really in getting the two teams to mix, you know, two different wards, they work very differently, different consultants, different specialties, different managers, different teams, lot of staff on set shifts. Erm, my staff don’t work set shifts, so the expectations of the staff … (MAN-CO-22)*

Another of the major challenges to implementing ERAS reported across the three specialities was patients being admitted via the intensive care unit (ICU) where the culture was for patients to not be provided with early nutrition, thus delaying ERAS implementation:

*…when the patients want they can have something to drink in recovery. Er, recovery generally aren’t that happy with that but we’re happy with that, but as soon as they get out to the ward they can eat and drink. Er, we find that falls down if they go to HD or ICU where there is not the, that same philosophy to get patients eating and drinking earlier. (SUR/ANS-TH-15)*

#### Spread

##### Within the hospital and beyond

Participants reported that the implementation of the programme within thoracics had been particularly successful. Whilst staff within this department considered themselves as instrumental in educating and spreading ERAS practice to the same speciality in other hospitals, those clinicians tasked with rolling the programme out reported capturing the successes in thoracics and transmitting it to other specialities within the same hospital as challenging:

*It can be very difficult to appreciate within the trust …how we’re perceived by the outside world, because we are seen as a beacon nationally and internationally in enhanced recovery, but sometimes you get the impression within the Trust that we’re just seen as annoying because we keep wanting to implement change. (SUR/ANS-TH-15)*

*Our main challenge has been moving on outside of thoracic surgery to other specialities, and we have had some successes, and we’ve had some failures. (SUR/ANS-TH-16)*

An ERAS lead described how although they had particular links within their own department which made it easier to engage and mobilise staff, they felt an outsider in other specialties and so had less leverage to request change:

*The stumbling block is when everybody says, “Well, you’re the lead now. Go and do it in other specialties.” But that’s not my specialty … I don’t have the same ability to change people’s opinions of what we should do, because they don’t see me on a day-to-day basis, doing the clinical work. (SUR/ANS-TH-16)*

##### Segmental approach

Due to the diversity of patients within the head and neck speciality, participants reported that separate ERAS pathways were being established for different surgical procedures. At the time of fieldwork only one procedural pathway had been devised, so there was limited depth of implementation within this speciality. Most staff on this ward reported that this segmental approach to implementation had been challenging, as the infrequency of patients on an ERAS pathway meant that the programme wasn’t part of ward culture, impacting on the level of staff enthusiasm and engagement and making it difficult to distinguish those patients on an ERAS pathway:

*I think, even now, people aren’t great with it ‘cause they were only doing flap patients. So, we’ll have one and then nothing … There’s a blob next to the board [to denote ERAS], but I don’t think anyone is really taking it onboard. I think, I’ve tried to, sort of, with the Senior Nurse, tell [them] what it’s about. But then you find the paperwork, it might be completed, but then it’s a bit halfhearted. (NUR/AHP-HN-10)*

Participants also reported that the irregularity of its use brought up difficulties of staff remembering to use the programme, and identifying those patients that were on the programme:

*So then because it’s not a common thing … people forget it so you can have patients that don’t come for three weeks and there’s no enhanced recovery at all, and then another one comes and it’s on enhanced recovery and then they go, “What does that mean again?” (NUR/AHP-HN-12)*

The limited number of patients on ERAS was also considered challenging because it reduced the amount of feedback about the programme. One individual stated, “*…if there was more patients, I could see more change*.” (NUR/AHP-HN-10). Staff perceived that a greater number of patients on an ERAS pathway would facilitate implementation embedding it in day-to-day practice:

*It should be that you walk onto the head and neck ward, and everybody is on the enhanced recovery programme. I think one of the things that we’ve got wrong, as I say, is you almost have this guy in different coloured pyjamas with a hat on, that is the enhanced recovery patient … (*SUR/ANS-HN-7*)*

*I think if we roll it out for more patients, we’ll see the staff using it better. But at the moment I can’t really honestly say that it’s been that useful… (NUR/AHP-HN-10)*

#### Health issue

Some individuals noted that it was particularly challenging to implement ERAS for the head and neck speciality:

*I was keen that head and neck didn’t get left behind really, erm, and it’s a bit difficult because they’re a difficult group because what people are fixated on is length of stay, and some of the things that cause head and neck patients to stay in are not easily changeable. Things like their tracheostomies, erm, but I still thought that, you know, that the quality aspects and the rest of ERAS would be very applicable, erm, to our patients and, er slightly challenging for some of them (Laughter).* (SUR/ANS-HN-8)

This was in contrast to the characteristics of the thoracic population which was felt to better suit ERAS implementation:

*Particularly with thoracic patients; you need to get them up and about and Enhanced Recovery is about getting the patients up and about. Erm, so I think it just suited that population really, really well. (NUR/AHP-TH-19)*

The type of speciality challenged the early post-operative feeding element of ERAS. The site of operation in the thoracics specialty allowed for minimal consideration when adopting the early feeding element of ERAS as the digestive tract is less affected. In contrast, health care professionals in the head and neck specialty felt they had greater complexities (e.g. anastomosis) to consider before feeding their patients post-surgery.

*Of course, all these other things and different surgical specialties, it has different impacts, so that for a thoracic surgeon it’s less likely to be a problem, feeding is less likely to be a problem, whereas, if you’ve got an anastomosis through which food is likely to go, then that is worrying…(SUR/ANS-HN-6)*

### Reflective monitoring – Gaining ownership of the ERAS programme

#### Data - collection and feedback

Participants reported that ERAS-related data collection and subsequent monthly feedback facilitated implementation as it highlighted areas that needed improvement. Supportive data and relevant feedback was therefore considered key to sustaining ERAS efforts:

*Because we could get some realistic data month on month back about length of stay, about patient experience, about compliance with nutritional drinks, about every kind of aspect of the enhanced recovery programme. And that started to focus it and really embed it into practice. (NUR/AHP-CO-24)*

Data collectors were considered to be crucial in this process, and participants reported that it was challenging when this resource wasn’t available (due to project specific funding ending and posts not being filled), and data analysis tasks fell to them:

*I would like to be able to produce the feedback for people ‘cause people have put a lot of effort in … on the other hand, you know, I don’t have the time to personally go and trawl through and get all that data. (SUR/ANS-HN-8)*

A few participants reported having used locally-generated data to challenge embedded behaviours and encourage practice to be in line with the ERAS programme:

*So we wanted to change the pain protocol and put them on a different style of pain relief, and that was hugely difficult to do in the anaesthetic department. It was really difficult, and the only way we could do it is by some of us doing it and auditing our data, getting a pain team involved and developing a protocol and comparing our data, new and old, and slowly, over two to three years, most people have moved away from epidurals. But that was a big challenge, and you can’t, to an anaesthetist who’s trained for 15 years of his life, go up to them and say, “You will stop using epidurals now. You will do it this way.” Because the evidence base doesn’t necessarily exist. (SUR/ANS-TH-16)*

#### Adapting the care pathway

Participants reported the necessity of customising and adapting generic guidelines to meet their patients’ needs when designing care pathways:

*The way that we developed the pathway was to look at the generic enhanced recovery pathway produced by the NHS in their document from 2010 and I took out parts of that pathway which were truly generic, erm so applicable to all specialties including ours, erm, so used those … as the skeleton of the, pathway, and then took other … evidence-based measures from the literature and also from what we were already doing, and incorporated them into our pathway. (SUR/ANS-TH-15)*

Staff reported that numerous subsequent adjustments or ‘tweaks’ to the pathway had been necessary, as had the conduct of audits to prove programme effectiveness:

*We’ve tweaked um, the laxatives. That’s a big part of it. Um, the regime we started on wasn’t that effective so we changed it a bit. We spoke with pharmacists and people like that, um, to get to where we are now. (NUR/AHP-TH-17)*

## Discussion

This study offered an in-depth exploration into both the facilitating factors and challenges of introducing ERAS into routine practice in various specialities. By focussing particularly on the nutritional aspects of ERAS it provided integral contextual information that may be useful for programme implementation [6, 26–28].

The present study, as others before [8, 9, 11], found that having standardised guidelines based on best evidence facilitated the adoption of an ERAS programme. Protocols were used as a tool both to remind and to challenge others’ practice. Ament et al. [10] reported a similar finding, where the guideline could be referred to if others deviated from the ERAS programme. However, the present study also found that healthcare professionals reported that some degree of flexibility was needed to ensure that individual patient needs could be met. Those implementing an ERAS programme should be aware of the potential struggle healthcare professionals’ face in balancing the benefits of following strict protocols with the desire to provide individualised care.

The health care professionals interviewed believed that for ERAS to be sustainable it needed to become standard care. This is in keeping with Gotlib Conn et al.’s [12] study, which suggested an important enabler to ERAS implementation was the ‘normalization of ERAS as everyday practice’. They suggest that if the elements are to become standard practice, the visibility of the programme needs to diminish without the programme ‘disappearing’. The present study found that incorporation of a practice into a routine activity, (e.g. provision of Fortisip drinks incorporated into the drug round and standardised), helped embed that aspect of ERAS. The findings also suggest that a segmental approach to implementation, as employed in the head and neck speciality, presented challenges to standardisation and embedding of the programme.

This study found key facilitators similar to previous studies conducted outside of the UK such as: teamwork and collaboration [7, 9, 11, 12]; meetings to provide opportunities for programme development, networking, and clarification [7, 12]; patient involvement and education [7–9, 29]; and staff education [9–11], which confirms their importance in various contexts, both in the UK and in specialities other than colorectal.

In line with previous qualitative research in this area [7–9, 12], leadership was considered to be an important facilitating factor to programme implementation. In the present study, the drive from leadership on the ward from either nurses or consultants was considered essential. This suggests that engaging leadership across multiple stakeholder groups is integral to programme implementation. It is commonly reported in the literature that the time and energy invested in a programme by a clinical ‘champion’ is central to successful programme implementation [30]. However, the present study highlighted the fragility of this ‘centre pin’ approach, as the over-reliance on one individual threatened implementation. The multimodal care approach of ERAS requires diverse professional groups to cooperate across multiple clinical boundaries. The findings suggest that having several champions who can operate across the differing disciplines to drive the programme forward may be a stronger leadership approach.

This study also revealed the importance of adapting ERAS guidelines to meet the needs of different specialities. The differing wards reported customising aspects of the programme to fit with their own processes and preferences. Modifying the programme for the local context is important for implementation success [12]. Denis et al. [31] conceptualised improvement programmes as having a “hard core” (elements that are well-defined and relatively fixed) and a “soft periphery” (elements that are less clear and more open to alteration). They suggest that “playing within the soft periphery” of a programme can have benefits, as programmes that may not have fit within a local context are made viable. However, this may be risky as negotiation in this way may dilute the elements of the programme, reducing the value in ways that affect patient care. Therefore, although our study suggests ERAS programmes need to be responsive to different clinical contexts through adaptation, it might be useful to establish the “hard core” non-negotiable elements to aid implementation.

Data feedback was felt to be integral to improvement efforts. It was used to motivate changes, highlight areas requiring work and made implementation successes visible. Monitoring, and data feedback is a widely discussed activity to maintain programme visibility [9, 10, 12] and this study suggests that adequate resources should be made available to sustain this valued process.

Although managers reported that they believed in ERAS and wanted its adoption throughout the hospital, this was not translated to clinical staff who perceived a lack of management support and commitment. This was reported as a challenge to implementing ERAS, an issue that has been previously noted [12]. Support was viewed as critical at times when improvement efforts were slowed by staffing issues. Outside of the ERAS literature, advocacy from management is known to be key for successful programme implementation [32]. The findings of this study suggest that every effort should be made to ensure managers support is made evident at ward level.

The present study found that healthcare professionals felt that too much information was given to patients and ERAS material was forgotten. This finding is in line with Alawadi et al. [11] who found that both the patient and provider were dissatisfied with patient education, and with Short et al. [33] who recently found that within an ERAS context, patients reported that the information they were provided was too general, repetitive and contradictory. As well as method of delivery [34] our study advocates that information provision, in terms of content and timing, requires further attention.

Other challenges to ERAS implementation were also found to be important across specialities in this UK hospital. These included individual-level resistance [7, 10, 12], overcoming traditional perceptions of perioperative care [7], a lack of resources in terms of staffing, space and time [9, 11], gaining buy-in from others and sustaining this engagement [9, 10, 12], and maintaining implementation post-project [12].

However, some of the identified challenges have not been previously reported within the qualitative ERAS literature but have been considered within the wider implementation field [35]. For example, programme spread was a challenge. More specifically, clinicians and managers reported spreading practice within the same hospital to other specialities as more challenging than to the same specialities in other hospitals. Programme implementation is tested when interventions are expected to diffuse on their own and readily transfer from one context to another [36]. The present study highlights the sensitivity of disseminating information, and the need for clear messages of change emanating from someone with authority and credibility *within* a speciality, and suggests profession-specific similarities are important for spread.

When rolling out the programme within a hospital the variability between specialities should be considered. More planning and resources may need to be provided to specialties that are considered to have greater variety and complexity within their patient group. The diversity within the head and neck speciality, and the decision to design different pathways to suit the various patient groups, meant that ERAS was only partially implemented. Implementation on a select few could be considered as piloting and is useful if set up appropriately [37]. However, the present study suggests that those deciding to limit the depth of ERAS implementation should be mindful of the possible challenges connected to this approach, such as staff forgetting to use the programme.

Transitions on the patient’s journey could strengthen or undermine ERAS implementation. Staff within the study by Alawadi et al. [11] predicted that managing implementation across departments may be challenging. This study affirms such apprehensions. Whilst the pre-operative assessment unit was considered an integral stage, patients admitted via ICU, and the difference in culture in this setting, were challenging for implementation.

Of the challenges and facilitating factors identified, some were particularly relevant to the nutritional component of ERAS. As previously cited [9, 11] alignment with evidence based practice was found to facilitate implementation. Past research reported that surgeons and anaesthetists were likely to resist adoption of the carbohydrate loading, early feeding and oral nutritional supplementation due to limited or weak supporting evidence. Although the early feeding element met with some resistance, healthcare professionals in the present study were supportive of the nutritional elements, and where the evidence was weak in a specialty, they looked to others with more evidence. Lyon et al. [8] reported that unrealistic post-surgery eating expectations could challenge the compliance to the nutritional elements. This is in accordance with the present study which found patient education and involvement was a key facilitator to encourage consumption of oral nutritional supplement drinks. It further suggests that ERAS diaries maybe a useful tool to engage patients to take an active role in their care. The palatability of the nutritional supplement drinks was the most frequently discussed challenge to the implementation of oral nutritional supplements. Future work is needed into how the drinks could be made more acceptable to patients.

One potential strategy to drive ERAS implementation forward, and reduce some of the identified barriers, is the use of community-based approaches. In contrast to traditional approaches to change (e.g. hierarchical), this approach to healthcare improvement is growing in appeal due to low-cost knowledge transfer and behaviour change through peer influence and ‘bottom-up’ participation. When implementing ERAS, the clinical community model may be favourable to other network based approaches (e.g. communities of practice and collaboratives) as it offers an improvement architecture that goes beyond just knowledge-sharing [38]. It creates an environment for change through the use of key features such as a ‘strong vertically-integrating core’ and ‘horizontal links’ between members [39].

The strength of this study is that, to our knowledge, it is the first study to explore the facilitating factors and challenges to the implementation of an ERAS programme from a UK context, and in specialisms other than colorectal surgery. Research that identifies possible factors that could potentially influence implementation in differing contexts is essential to help advance and spread improvement [4]. Another strength is the broad range of stakeholders included in the study, including allied healthcare professionals and managers. This is important because the ERAS programme is a complex intervention requiring support from a number of stakeholders [7]. The voice of the various healthcare providers has previously been difficult to discern with many studies only capturing the perception or practice of the surgeon or anaesthetist [13, 40–44]. A potential limitation is that those who agreed to take part in the study were more familiar with and/or more amenable to the ERAS programme, however both positive and negative aspects of ERAS implementation were expressed by participants. A further limitation is the study was based in one hospital, and therefore results may not necessarily be generalizable to others. However it is important to begin to build up an evidence base of contextual factors that influence implementation in the UK and amongst specialities other than colorectal. Although statistical generalisation cannot occur, analytical generalisation is available as those utilising these findings can make their own decisions about similarities and differences of their context to the one documented here [4].

## Conclusion

This study identified challenges and facilitating factors to ERAS program implementation from the perspective of healthcare professionals. The findings presented here provide useful contextual qualitative information from diverse surgical specialities to inform healthcare providers when implementing ERAS in practice. Addressing the challenges (e.g. keeping ERAS visible and spreading the programme) and utilising the facilitating factors (e.g. cohesive, visible leadership amongst surgeons and nurses) identified in this study, could speed up the rate at which ERAS is adopted, implemented and embedded.

## Abbreviations

CQUIN: Commissioning for Quality and Innovation
ERAS: Enhanced Recovery After Surgery
NPT: Normalization Process Theory
